# CD4^hi^CD8^low^ Double-Positive T Cells Are Associated with Graft Rejection in a Nonhuman Primate Model of Islet Transplantation

**DOI:** 10.1155/2018/3861079

**Published:** 2018-07-10

**Authors:** Yun Jung Choi, Hi-Jung Park, Hye Jin Park, Kyeong Cheon Jung, Jae-Il Lee

**Affiliations:** ^1^Graduate Course of Translational Medicine, Seoul National University College of Medicine, Seoul 03080, Republic of Korea; ^2^Transplantation Research Institute, Seoul National University Medical Research Center, Seoul 03080, Republic of Korea; ^3^Department of Pathology, Seoul National University College of Medicine, Seoul 03080, Republic of Korea; ^4^Department of Medicine, Seoul National University College of Medicine, Seoul 03080, Republic of Korea

## Abstract

Peripheral CD4/CD8 double-positive (DP) T cells are associated with autoimmune disorders, cancer, and viral infection. However, the relationship between organ transplantation and DP T cells is unclear. Here, we examined the functional characteristics of peripheral DP T cells and analyzed their significance with respect to islet graft rejection in a nonhuman primate model of islet transplantation. DP T cells were functionally equivalent to conventional CD4 and CD8 T cells in terms of helper and cytotoxic activity, respectively. DP T cells expressed high levels of CXCR5 and PD-1 and secreted IFN-*γ*, IL-4, and IL-21 in amounts equivalent to those secreted by CD4 or CD8 T cells; also, they produced large amounts of granzyme B and perforin. In addition, under steady-state conditions, DP T cells expressed eomesodermin (Eomes) and promyelocytic leukemia zinc finger (PLZF) proteins, both of which act as transcription factors in innate/memory-like T cells. The number of peripheral DP T cells in the islet transplantation model was high in the group that experienced graft rejection; this was not the case in the long-term survival group. Interestingly, numbers of effector memory T cells (TEM) within the DP T cell population increased significantly during islet graft rejection, as did those of TEM within the cytotoxic CD8 T cells. Furthermore, the most conspicuous of which was the increase of CD4^hi^CD8^low^ T cell subpopulation at that point. Taken together, the data suggest that peripheral DP T cells showing an innate/memory-like phenotype have both helper and cytotoxic activity *in vitro* and that they may act as a novel biomarker for graft rejection after islet transplantation.

## 1. Introduction

Studies conducted over the last two decades show that the peripheral blood of both humans [1–3] and animals [4] contains a substantial number of CD4/CD8 double-positive (DP) T cells. DP T cells represent one of the T cell developmental stages within the thymus; however, unlike thymic DP T cells, peripheral DP T cells display varying levels of coreceptor expression, a memory phenotype, and none of the markers typical of recent thymic emigrants [5–7]. Therefore, peripheral DP T cells are defined as an extrathymic population [8].

Two hypotheses have been proposed to explain the developmental pathway for DP T cells. One is that positive thymic selection fails to delete both coreceptors; therefore, DP T cells easily pass through [6]. The other is that, under certain circumstances (i.e., disease), mature single-positive (SP) T cells might acquire another coreceptor, either CD4 or CD8, enabling it to secrete a variety of inflammatory cytokines [9–13]. As reported previously, peripheral DP T cells exhibit several characteristics, including a CD1b^−^CD4^+^CD8^low^ phenotype, expression of CD8*αα* homodimers, a resting memory phenotype, and share the same T cell receptor (TCR) V*β* with CD4 SP T cells [14, 15]. Peripheral DP T cells, if they were developed via unconventional pathways, might express unique features; examples include innate T cells or another distinct T cell lineage.

Recent reports show that promyelocytic leukemia zinc finger protein- (PLZF-) positive CD4 T cells generate eomesodermin- (Eomes-) positive thymic CD8^+^ T cells during thymic development [16–19]. Lee et al. reported that the memory-like CD8^+^ T cells expressing Eomes constitute another subset of innate T cells [20]. Peripheral DP T cells expressing phenotype markers typical of innate T cells may exhibit distinct characteristics depending on the peripheral environment.

Peripheral DP T cells play a helper function role during progression of autoimmune diseases such as thyroiditis [21], atopic dermatitis [22], systemic sclerosis [23], and rheumatoid arthritis (RA) [24]. In particular, Quandt et al. reported that DP T cells (mainly CD4^hi^CD8^low^) in RA seem to contribute to the inflammatory process by secreting cytokines such as IL-4, IL-21, and IFN-*γ* [11].

However, Zloza et al. reported that CD4^dim^CD8^bright^ T cells are an enriched antiviral subpopulation and recognize an antigen-specific target in HIV-positive patients [25, 26]. Sarrabayrouse et al. showed that DP T cells can play a suppressive role in metastatic colorectal cancer [27]. These conflicting reports suggest that the immunological functions of this cell population remain unclear.

Here, we examined the functional characteristics of peripheral DP T cells (e.g., expression of transcription factors, cytokines, and enzymes). Furthermore, we used a nonhuman primate islet transplantation model to examine whether peripheral DP T cells play a role in graft rejection.

## 2. Materials and Methods

### 2.1. Subjects

Adult naïve rhesus macaques (8 males and 15 females; age, 48 to 72 months; weight, 3.72 to 5.7 kg) were used for the study. After being imported from China, the animals remained in quarantine for 1 month, during which they were in good condition. Each monkey was housed in a single cage with access to biscuits (2050 Harlan, Teklad Diets, Madison, WI, USA) and some fresh fruits and vegetables. Access to water was unlimited. All animals were cared for in strict accordance with the National Institutes of Health Guide for the Care and Use of Laboratory Animals. This study was approved by the local Institutional Animal Care and Use Committee (IACUC) of Seoul National University Hospital (IACUC number: 14-0002-C2A0).

### 2.2. Samples

Heparin- or EDTA-anticoagulated whole blood was obtained from the monkeys, and cells were isolated for functional analysis and phenotyping. Peripheral blood mononuclear cells (PBMCs) were separated by density gradient centrifugation on Ficoll-Paque (GE Healthcare, Uppsala, Sweden). Isolation of lymphocytes from mesenteric lymph nodes (MLNs), the spleen, and the liver was performed after autopsy (*n* = 5). Tissues were minced into a single-cell suspension and then resuspended in RPMI 1640 medium supplemented with 10% FBS at 4°C.

### 2.3. Cell Sorting

To separate CD4 SP, CD8 SP, and DP T cells, PBMCs were stained with anti-CD4 and anti-CD8 antibodies and resuspended in PBS supplemented with 1% FBS. Cells were then sorted on a BD FACS Aria instrument (BD Biosciences, San Diego, CA, USA).

### 2.4. Flow Cytometry Analysis

Fluorochrome- or biotin-labeled human monoclonal antibodies specific for the following antigens were purchased from BD Biosciences (San Jose, CA, USA), eBioscience (San Diego, CA, USA), and BioLegend (San Diego, CA, USA): CD8*α* (SK1), CD4 (L200), CD3 (SP34-2), CD28 (CD28.2), CD95 (DX2), CD1b (SN13), CD8*β* (SIDI8BEE), HLA-DR (G46-6), CXCR5 (MU5UBEE), and PD-1 (EH12.2H7). Single-cell suspensions were labeled with the appropriate antibodies for 30 minutes at 4°C. For intracellular labeling, prepared cells were resuspended in a mixture of fixation and permeabilization buffers provided in the Foxp3 staining buffer kit (eBioscience, San Diego, CA, USA). Cells were labeled with antibodies specific for Eomes (BD Biosciences, San Jose, CA, USA) and PLZF (eBioscience, San Diego, CA, USA). Flow cytometry was performed using a FACS Calibur cytometer (BD Biosciences, Mountain View, CA, USA) and an LSR Fortessa (BD Biosciences, Mountain View, CA, USA). All data were analyzed using FlowJo software (TreeStar, Ashland, OR, USA).

### 2.5. Staining for Cytokine Analysis

For intracellular cytokine assay, peripheral CD4, CD8, and DP T cells from primate whole blood were sorted and then stimulated with 50 ng/mL phorbol 12-myristate 13-acetate (PMA) and 1.5 *μ*M ionomycin (Sigma-Aldrich, St Louis, MO, USA) and treated with 6.7 *μ*g/mL monensin (Sigma-Aldrich, St Louis, MO, USA) for 6 hours at 37°C in a CO_2_ incubator. Next, cells were washed with complete medium (RPMI/10% FBS) and resuspended in staining buffer (PBS, 0.5% BSA, and 0.5 mM EDTA). The cells were then fixed, permeabilized, and labeled with anti-IL-4 (BioLegend, San Diego, CA, USA), anti-IL-21 (BioLegend, San Diego, CA, USA), and anti-IFN-*γ* (BioLegend, San Diego, CA, USA) antibodies.

### 2.6. Staining of Granzyme B and Perforin

Peripheral CD4, CD8, and DP T cells were stimulated with PMA (50 ng/mL) and ionomycin (1.5 *μ*M) and treated with monensin (6.7 *μ*g/mL) for 6 hours at 37°C in a CO_2_ incubator. After culture, cells were washed with complete medium (RPMI/10% FBS) and resuspended in staining buffer (PBS, 0.5% BSA, and 0.5 mM EDTA). The cells were then fixed, permeabilized, and stained with anti-granzyme B (BioLegend, San Diego, CA, USA) and anti-perforin (Mabtech, Nacka Strand, Sweden) antibodies.

### 2.7. Analysis of DP T Cells in an Islet Transplantation Model

Monkeys were treated with streptozotocin (100–120 mg/kg i.v.; Sigma-Aldrich, Saint Louis, MO, USA) to induce diabetes and then transplanted with xenogeneic and allogenic islet cells intraportally, as described previously [28, 29]. Pig to monkey islet xenotransplantation was performed in six cases (Rm01, Rm02, Rm03, Rm04, Rm05, and Rm06) and monkey islet allotransplantation in the remaining two cases (Rm07 and Rm08). The DP T cell population in the peripheral blood was monitored before transplantation and on days 0, 3, 7, 14, and 28 posttransplantation. Counts were then undertaken monthly for up to 6 months and then every 2 months thereafter.

### 2.8. Statistical Analysis

All data were analyzed using the PRISM program (GraphPad Software Inc., La Jolla, CA, USA). For all cell analysis data, the significance of differences was determined by *t*-testing. Numbers of DP T cells in the blood of transplanted monkeys were analyzed by linear regression. A *p* value < 0.05 was considered significant.

## 3. Results

### 3.1. Peripheral DP T Cells Express Eomes and PLZF

**Figure 1:**
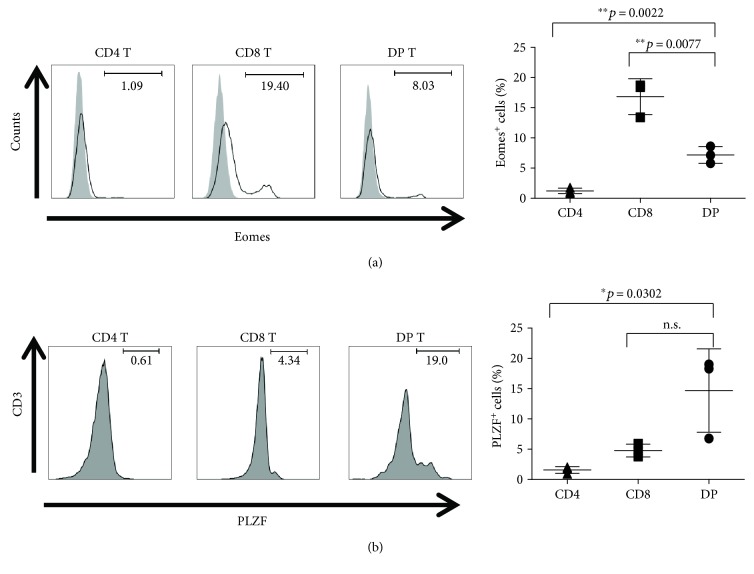
Expression of Eomes and PLZF in peripheral DP T cells. CD4, CD8, and DP T cells were stained with antibodies specific for (a) Eomes and (b) PLZF (*n* = 3; aged between 48 and 54 months). The histograms (left panels) show data from one representative experiment. Cumulative data are shown in the right panels. Data are expressed as the mean ± SD. Eomes: eomesodermin; PLZF: promyelocytic leukemia zinc finger protein. ^∗^ *p* < 0.05 and ^∗∗^ *p* < 0.01. n.s.: not significant.

To identify novel transcription factors involved in DP T cell development, we examined expression of Eomes and PLZF, which are expressed by T cells with innate immune properties [30]. A previous report showed that Eomes are expressed by CD8^+^ T cells but not by CD4^+^ T cells [31]. The results obtained herein agreed with this: Eomes was expressed mainly by CD8^+^ T cells in the peripheral blood of rhesus monkeys. However, DP T cells also expressed relatively high levels of Eomes, although less than those expressed by CD8^+^ T cells (Figure 1(a)).

PLZF expression is low or absent from conventional naïve, effector, or memory CD4^+^ and CD8^+^ T cells and from unconventional effector populations such as intraepithelial *γδ* and *αβ* lymphocytes [32].  Figure 1(b) shows that peripheral DP T cells expressed much higher levels of PLZF than CD4^+^ and CD8^+^ T cells. Thus, peripheral DP T cells express both Eomes and PLZF.

### 3.2. Distribution of DP T Cells in the Peripheral Blood, Lymphoid Tissues, and Liver

**Figure 2:**
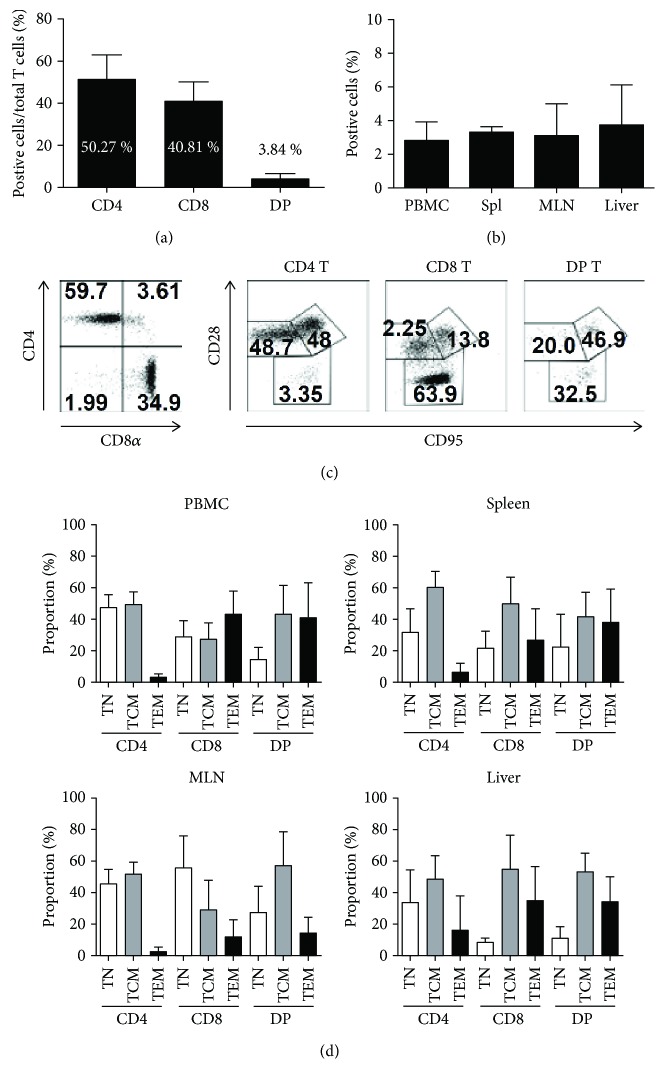
Percentage of DP T cells in peripheral blood and tissues isolated from rhesus monkeys. (a) The percentage of CD4, CD8, and DP T cells in the peripheral blood (*n* = 23; aged between 48 and 72 months) and (b) the percentage of DP T cells in the peripheral blood mononuclear cell population, spleen, MLNs, and liver (*n* = 5; aged between 48 and 54 months) of rhesus monkeys. (c and d) Phenotypic characterization of T cell subsets in peripheral blood and tissues from rhesus monkeys using antibodies specific for CD28 and CD95. Data are expressed as the mean ± SD. PBMC: peripheral blood mononuclear cells; MLNs: mesenteric lymph nodes; TN: naïve T cells; TCM: central memory T cells; TEM: effector memory T cells.

To examine the distribution of DP T cells in the periphery, we counted the numbers of DP T cells in the peripheral blood, liver, and lymphoid tissues (spleen and MLNs). The results are shown in Figures 2(a)–2(c). The percentages of CD4^+^ and CD8^+^ in total T cells were 50.27% and 40.81%, respectively, whereas the proportion of DP T cells was 3.84% (Figure 2(a)). The proportion of DP T cells in the spleen and MLNs was 3.31% and 3.13%, respectively, in total T cells. Of note, the proportion in the liver was 3.76%, which is comparable to that in the lymphoid tissues (Figure 2(b)).

To determine phenotypic properties of the DP T cells in the periphery, we stained cells with antibodies specific for CD28 and CD95 (Figure 2(c)). DP T cells in peripheral blood are characterized as having a resting memory phenotype [14, 33]. As expected, we confirmed this finding.  Figure 2(d) shows that most DP T cells in the peripheral blood, spleen, and liver expressed CD28^+^CD95^+^ (central memory) or CD28^−^CD95^+^ (effector memory) phenotypes. However, DP T cells in the lymph nodes expressed mainly naïve or central memory phenotypes.

### 3.3. DP T Cells Demonstrate Both Helper Function and Cytotoxic Activity In Vitro

**Figure 3:**
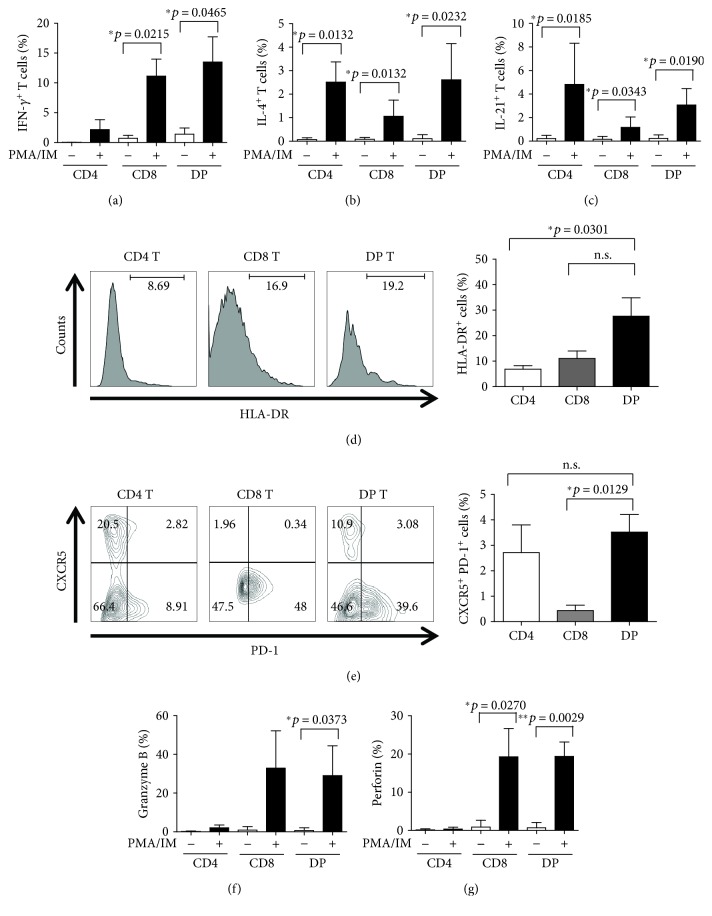
DP T cells show helper functions and cytotoxic activity *in vitro*. After sorting T cells, CD4, CD8, and DP T populations were stimulated with PMA/ionomycin and stained with antibodies specific for (a) IFN-*γ*, (b) IL-4, and (c) IL-21 (*n* = 5; aged between 48 and 54 months). The percentage of T cells positive for (d) HLA-DR and (e) CXCR5/PD-1 is indicated (*n* = 4; aged between 48 and 54 months). Sorted cells were stimulated with PMA/ionomycin and then stained with antibodies specific for (f) granzyme B and (g) perforin (*n* = 4; aged between 48 and 54 months). Data from one independent experiment (left panels) and cumulative data (right panel) are shown. Data are expressed as the mean ± SD. ^∗^ *p* < 0.05 and ^∗∗^ *p* < 0.01. n.s.: not significant.

To investigate the functional characteristics of peripheral DP T cells, we sorted DP T cells from peripheral blood and stimulated them with PMA/ionomycin. We then measured their capacity to produce IFN-*γ*, IL-4, and IL-21. The results showed that peripheral DP T cells produced a substantial amount of IFN-*γ*, more than that produced by CD8^+^ T cells (Figure 3(a)). DP T cells also produced IL-4 and IL-21 in amounts similar or equivalent to those produced by CD4^+^ T cells (Figure 3(b) and 3(c)). Furthermore, expression of HLA-DR, a T cell activation marker [34], was higher in DP T cells than in CD4^+^ or CD8^+^ T cells (Figure 3(d)). Peripheral DP T cells expressed equivalent levels of CXCR5 and PD-1, which are biomarkers for follicular helper T cells [35], when compared to CD4^+^ T cells (Figure 3(e)). Taken together, these data suggest that DP T cells play a helper role during innate or adaptive immune responses.

We also measured granzyme B and perforin, mainly expressed by cytotoxic CD8^+^ T lymphocytes, in each T cell subset. The results are shown in Figures 3(f) and 3(g). As expected, CD8 T cells produced large amounts of these enzymes upon stimulation with PMA/ionomycin. However, peripheral DP T cells also secreted both cytotoxic enzymes and at levels similar to those observed in CD8 T cells.

### 3.4. DP T Cells Are Markedly Increased in the Monkeys That Rejected Islet Grafts

**Figure 4:**
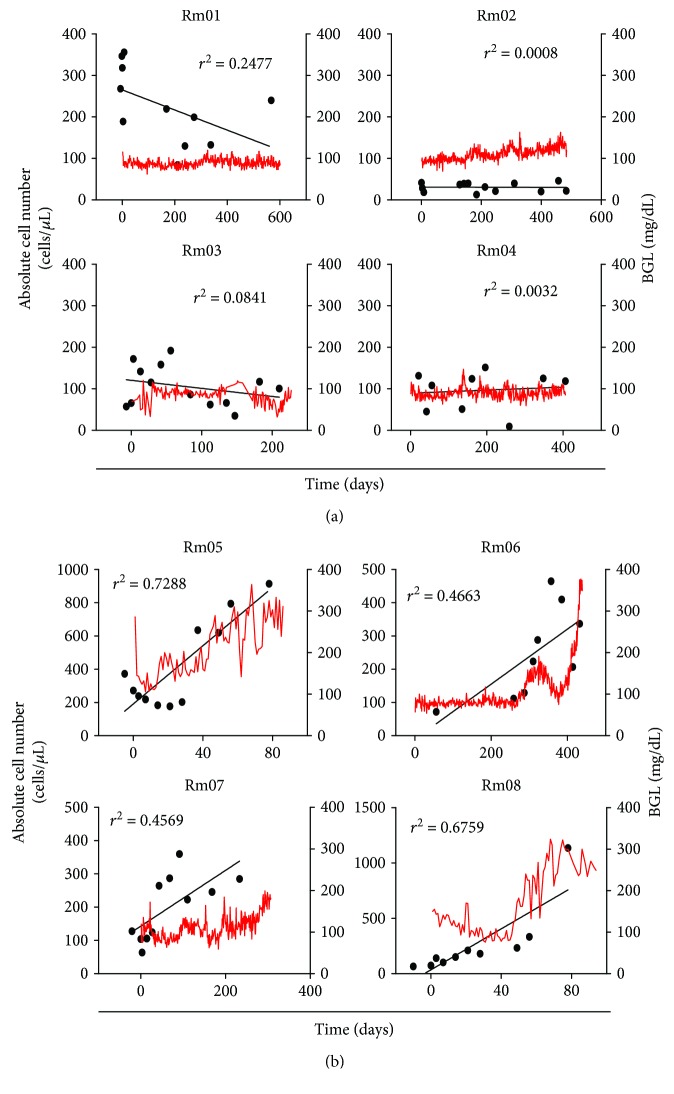
The number of peripheral DP T cells increased significantly in monkeys that rejected islet cell transplants. (a) The number of DP T cells in the peripheral blood of monkeys in which transplants survived did not change markedly (*n* = 4; aged between 54 and 72 months). (b) The number of DP T cells in the peripheral blood of monkeys that rejected the islet grafts increased significantly when compared with pretransplant levels (*n* = 4; aged between 54 and 72 months). BGL: blood glucose. Rm01, Rm02, Rm03, Rm04, Rm05, and Rm06 received islet xenografts, and Rm07 and Rm08 received allografts.

Next, we examined the *in vivo* role of DP T cells using an islet transplantation model, in which chronic inflammation may occur. Monkeys surviving long term (defined as maintenance of normoglycemia) after transplantation did not show a noticeable variation in the DP T cell population during the follow-up period (Figure 4(a)). By contrast, the number of DP T cells in the peripheral blood of monkeys that lost graft function (defined as a distinct increase in blood glucose levels and a reduction in C-peptide levels) was significantly higher (Figure 4(b)). This distinct increase in the DP population was observed in monkeys receiving either xenograft or allograft.

**Figure 5:**
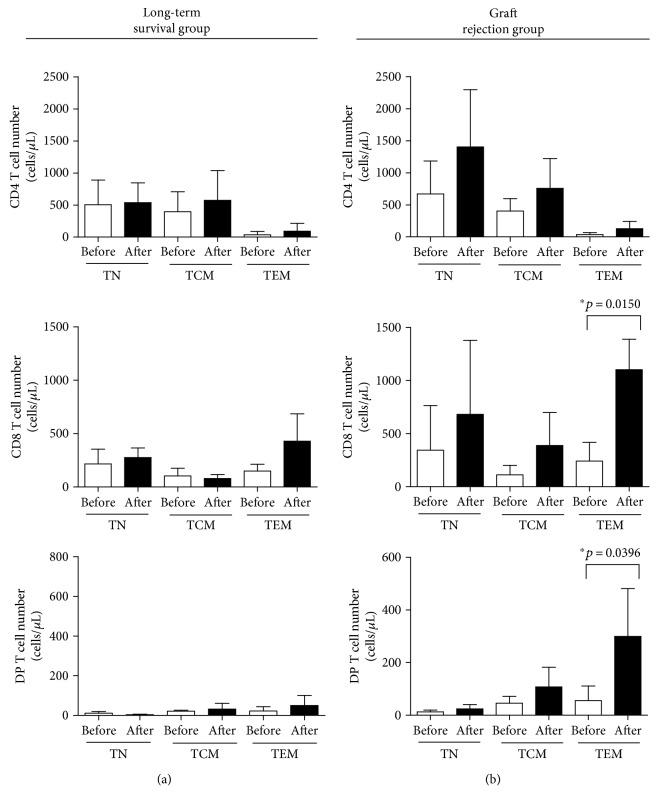
The TEM subpopulation of DP T cells in monkeys that rejected grafts increased markedly, as did the TEM subpopulation of CD8 cells. The subpopulations of CD4, CD8, and DP T cells were examined in (a) the long-term graft survival group (*n* = 4; age, 54–72 months) and (b) the graft rejection group (*n* = 4; aged between 54 and 72 months). Data are expressed as the mean ± SD. ^∗^ *p* < 0.05. TN: naïve T cells; TCM: central memory T cells; TEM: effector memory T cells.

Analysis of changes in T cell subsets before and after islet transplantation (Figures 5(a) and 5(b)) revealed a significant increase in the CD8 T cell subset and DP T cell subset in animals that rejected grafts (Figure 5(b)). In particular, the effector memory subpopulation of DP T cells increased (as did that of CD8 T cells).

**Figure 6:**
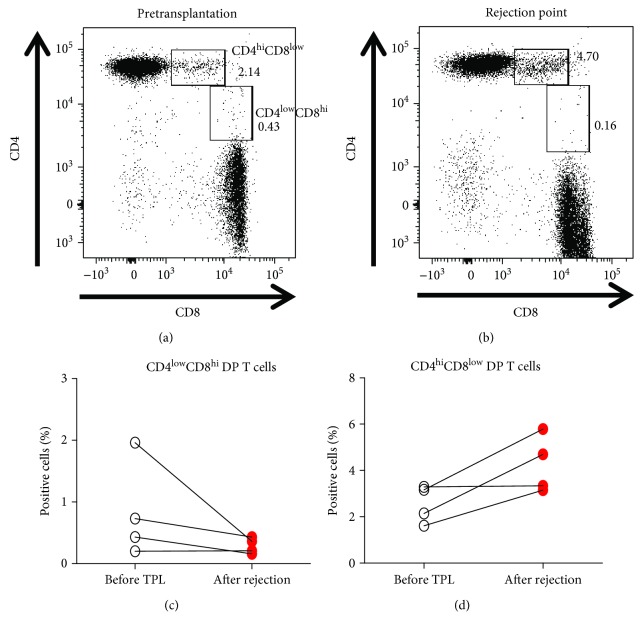
The CD4^hi^CD8^low^ DP T cell population increased markedly in the graft rejection group. Subpopulations of DP T cells were analyzed pretransplantation (a) and at the point of rejection (b). CD4^low^CD8^hi^ DP T cells (c) and CD4^hi^CD8^low^ DP T cells (d) pretransplantation were compared with those at the point of rejection. TPL: transplantation.

Two subsets of peripheral DP T cells have been reported based on the level of CD4 and CD8 expression, which may account for differences in DP T cell function [36]. Therefore, we analyzed our data further by gating on CD4^hi^CD8^low^ and CD4^low^CD8^hi^ subpopulations of DP T cells (Figures 6(a) and 6(b)). We found that the percentage of CD4^low^CD8^hi^ DP T cells either fell or remained unchanged after graft rejection (Figure 6(c)), whereas the percentage of CD4^hi^CD8^low^ DP T cells increased (Figure 6(d)).

## 4. Discussion

Studies have examined CD4/CD8 DP T cells with respect to their function and relevance to various disorders [37–41]. DP T cells can be divided into multiple populations depending on the level of CD4 and CD8 coexpression [7]. Therefore, they are able to possess polyfunctional features. Peripheral DP T cells have been defined as an extrathymic population [33], which is supported by results presented herein: peripheral DP T cells expressed a CD8*αα* homodimer but did not express CD1b molecules on their surface (Supplemental Figure 1).

Recently, Park et al. reported that some T cells with a memory-like phenotype in the thymus respond immediately to antigenic stimulation [42]. These T cells include NKT cells, PLZF^+^ T-T CD4 (or T-CD4) T cells, H2-M3-specific T cells, mucosal-associated invariant T (MAIT) cells, CD8*αα* ^+^ intraepithelial T cells, Eomes^+^ natural Th1 cells, and innate CD8 T cells expressing Eomes [42–44]. Jacomet et al. reported strong evidence for the presence of innate-like CD8 T cells in humans and showed that they are Eomes positive [45]. In addition to Eomes, PLZF is a transcription factor necessary for development of invariant NKT (iNKT) cells and human peripheral MAIT cells [32, 43]. In particular, PLZF^+^ innate T cells allow both effector and regulatory T cells to be activated in the thymus prior to their exit to the periphery [16–18].

The results presented herein show that the proportion of Eomes^+^ DP T cells in the peripheral blood of rhesus monkeys was as high as 7.18%, although this was lower than that of CD8 T cells (16.83%). In addition, the percentage of PLZF^+^ T cells within the peripheral DP T cell population was higher than that in the CD4 or CD8 T cell populations. Considering that peripheral DP T cells expressed higher levels of Eomes and PLZF than CD4 or CD8 T cells, we presume that they have innate and memory properties. Recently, White et al. showed that memory phenotype CD8^+^ T cells are present in mouse and human in substantial numbers (these were labeled innate memory T cells as they had not been exposed to antigen); this subset is poised to produce IFN-*γ* when it comes into contact with a cognate antigen [46]. Further studies are needed before firm conclusions about the functional properties and potential benefits of DP T cells can be drawn; it is likely that they possess innate-like behaviors not displayed by conventional *αβ* T cells with respect to cytokine production and transcription factor expression.

The results of cytokine analysis showed that the cytokine profile of DP T cells was consistent with cellular functions observed for DP T cells isolated from RA patients; peripheral DP T cells isolated from monkeys produced IFN-*γ*, IL-4, and IL-21 in response to mitogen stimulation. In addition, expression of CXCR5 and PD-1, which are expressed in follicular T cells that help B cells to produce antibodies [47–49], was equivalent to that observed in CD4 T cells. These data suggest that peripheral DP T cells have an inducer function, which may help them to communicate with other immune cells during an immune response. On the other hand, peripheral DP T cells generated as much granzyme B and perforin as CD8 T cells *in vitro*, confirming data published by Nam et al. [15]. Thus, peripheral DP T cells display helper/inducer functions (like CD4 T cells) and cytotoxic/cytolysis activity (like CD8 T cells), at least *in vitro*.

The liver is a unique anatomical and immunological site in which antigen-rich blood from the gastrointestinal tract is passed through a network of sinusoids and scanned by antigen-presenting cells and lymphocytes [50]. Here, we found that the liver of monkeys harbored a substantial number of DP T cells (3.76%), similar to peripheral blood and lymphoid organs. A majority of these cells (53.20%) expressed a central memory phenotype. In addition to conventional CD4 or CD8 T cells, therefore, circulating DP T cells may readily bind antigens displayed by endothelial cells, Kupffer cells, and dendritic cells in sinusoids, thereby helping facilitate a significant immune response (i.e., antigen-dependent or antigen-independent cytokine production and lytic activity).

Transplantation of islet cells into rhesus monkeys via the portal vein revealed a marked increase in effector memory T (TEM) DP T cells during graft rejection (much like the increase in TEM CD8 T cells). Furthermore, analysis of CD4^hi^CD8^low^ and CD4^low^CD8^hi^ subpopulations of DP T cells revealed a marked increase in CD4^hi^CD8^low^ DP T cells in the graft rejection group. Waschbisch et al. reported no marked difference between these two subpopulations in healthy humans and patients with multiple sclerosis (a chronic inflammatory condition) [51]. However, Nascimbeni et al. reported that high numbers of CD4^hi^CD8^low^ DP T cells were observed in patients chronically infected with hepatitis C virus [36]. By contrast, Sullivan et al. reported that CD4^dim^CD8^bright^ T cells are an activated phenotype of CD8^+^ T cells and described that CD4 upregulation on CD8^+^ T cells may have a critical impact on the pathogenesis of the HIV or any other CD4-utilizing viruses [52]. In this respect, the level of coexpression of CD4 and CD8 molecules on DP T cell seems to represent their critical features for a variety of environmental conditions. Regardless of chronic inflammatory conditions, eventually, CD4^hi^CD8^low^ cells of DP T population appear to have a distinct pattern that depends on antigen specificities. Further studies should determine whether CD4^hi^CD8^low^ DP T cells affect the survival of pancreatic islet grafts in the liver directly or indirectly. Furthermore, since NKT cells can do masking [7], the lineage and functional potential analysis of each individual subpopulation of CD4^+^CD8^+^ T cells may need to be analyzed.

## 5. Conclusion

Peripheral DP T cells derived from rhesus monkeys express an innate memory phenotype and have two different functions *in vitro*: a helper function and a cytotoxic function. In addition, the results from the islet transplantation model suggest that this cell population may be an important biomarker (along with CD8 T cells) of graft rejection.

## Data Availability

The data used to support the findings of this study are available from the corresponding author upon request.

## Supplementary Materials

Supplementary Materials Supplementary Figure 1: the peripheral DP T cells differ from thymic DP T cells.
