# Supplementary material for: CD4hiCD8low Double-Positive T Cells Are Associated with Graft Rejection in a Nonhuman Primate Model of Islet Transplantation
Source: J Immunol Res. 2018 Jul 10;2018:3861079. doi: 10.1155/2018/3861079 (PMC6079492; doi:10.1155/2018/3861079)
Supplement: Supplementary Materials — Supplementary Figure 1: the peripheral DP T cells differ from thymic DP T cells. [file 3861079.f1.pptx]

## Slide 1
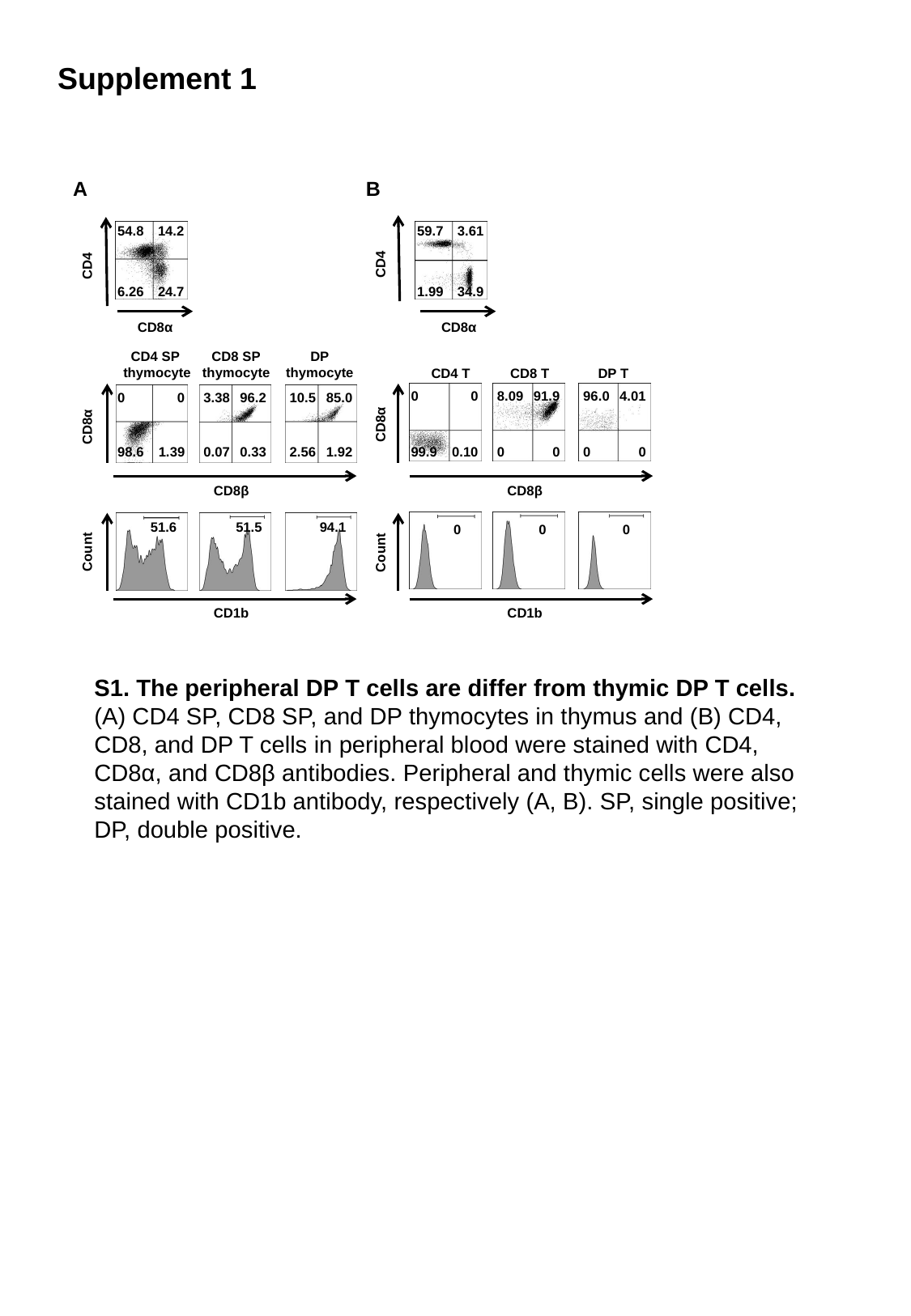

Supplement 1
A
B
54.8
14.2
CD4
6.26
24.7
CD8α
CD4 SP
thymocyte
CD8 SP
thymocyte
DP
thymocyte
0
0
98.6
1.39
3.38
96.2
0.07
0.33
10.5
85.0
2.56
1.92
CD8α
CD8β
51.6
51.5
94.1
Count
CD1b
59.7
3.61
CD4
1.99
34.9
CD8α
CD4 T
CD8 T
DP T
0
0
99.9
0.10
8.09
91.9
0
0
96.0
4.01
CD8α
0
0
CD8β
0
0
0
Count
CD1b
S1. The peripheral DP T cells are differ from thymic DP T cells. (A) CD4 SP, CD8 SP, and DP thymocytes in thymus and (B) CD4, CD8, and DP T cells in peripheral blood were stained with CD4, CD8α, and CD8β antibodies. Peripheral and thymic cells were also stained with CD1b antibody, respectively (A, B). SP, single positive; DP, double positive.
